# CYP epoxygenase metabolites of docosahexaenoic acid protect HL-1 cardiac cells against LPS-induced cytotoxicity through SIRT1

**DOI:** 10.1038/cddiscovery.2015.54

**Published:** 2015-11-23

**Authors:** V Samokhvalov, K L Jamieson, J Vriend, S Quan, J M Seubert

**Affiliations:** 1 Faculty of Pharmacy and Pharmaceutical Sciences, University of Alberta, Edmonton, AB, Canada; 2 Department of Chemistry and Pharmaceutical Sciences, Faculty of Sciences, VU University, Amsterdam, The Netherlands; 3 Department of Pharmacology, Faculty of Medicine, University of Alberta, Edmonton, AB, Canada

## Abstract

Bacterial LPS is an environmental toxin capable of promoting various cardiac complications. Current evidence suggests that LPS-induced myocardial dysfunction emerges as a consequence of compromised quality of cardiac mitochondria. Docosahexaenoic acid (DHA, 22:6n3) is an n-3 polyunsaturated fatty acid (PUFA), which produces a broad spectrum of intrinsic physiological effects including regulation of cell survival and death mechanisms. Although, numerous studies revealed fundamentally beneficial effects of DHA on cardiovascular system, it remains unknown whether these effects were produced by DHA or one of its possibly more potent metabolites. Emerging evidence indicates that cytochrome *P*450 (CYP) epoxygenase metabolites of DHA, epoxydocosapentaenoic acids (EDPs), produce more potent biological activity compared to its precursor DHA. In this study, we investigated whether DHA and its metabolite 19,20-EDP could protect HL-1 cardiac cells against LPS-induced cytotoxicity. We provide evidence that exogenously added or DHA-derived EDPs promote mitochondrial biogenesis and function in HL-1 cardiac cells. Our results illustrate the CYP epoxygenase metabolite of DHA, 19,20-EDP, confers extensive protection to HL-1 cardiac cells against LPS-induced cytotoxicity via activation of SIRT1.

## Introduction

Cardiovascular disease remains a leading cause of morbidity and mortality worldwide. Although many differing factors contribute to the etiology, recent data suggest the bacterial endotoxin, lipopolysaccharide (LPS) has a significant role in causing numerous cardiovascular complications.^1^ Adverse effects of LPS have been observed at concentrations (0.2 ng/m^3^) below those responsible for sepsis (e.g., endotoxemia).^2^ As such, the impact of increased environmental exposure to low LPS concentrations as a factor causing the pathogenesis of cardiovascular complications is largely underestimated.^3^ LPS is ubiquitously found in particulate matter, air pollution and numerous work environments as farms, research laboratories and waste management facilities. Current models postulate LPS-induced cardiotoxicity occurs as a direct result of inflammatory injury of mitochondria promoting cell death.^4,5^ However, the precise cellular and molecular mechanisms underlying these events remain poorly understood.

Long-chain n-3 polyunsaturated fatty acids (PUFAs), such as docosahexaenoic acid (DHA) are obtained from dietary sources and produce a broad spectrum of biological effects in both cell culture and animal models.^6^ Evidence suggests the risk of coronary heart disease is reduced with increased consumption of dietary n-3 PUFAs.^7^ Specific effects of DHA include improved cardiac and endothelial cell function, reduced inflammation, preserved cardiac mitochondrial function and reduced ischemia-reperfusion injury.^6–12^ DHA can be metabolized by cyclooxygenases (COX), lipoxygenases (LOX) and cytochrome *P*450 (CYP) enzymes to a vast array of biologically active lipid mediators.^8,9^ CYP epoxygenases add oxygen across one of the four double bonds of DHA to generate three-membered ethers known as epoxides. There are six regioisomeric metabolites termed epoxydocosapentaenoic acids (4,5-, 7,8-, 10,11-, 13,14-, 16,17- and 19,20-EDP). The predominant degradative pathway of EDPs is the formation of inactive vicinal diol compounds by soluble epoxide hydrolase (sEH).^9^ Recent studies demonstrated EDPs act as potent endogenous regulators of angiogenesis, RAAS, autophagy and insulin-dependent pathways.^10^ We recently demonstrated that EDPs are much more potent molecules compared to their precursor DHA.^11^

Although the concept that EDPs regulate key biological processes is novel, our understanding of their mechanism(s) of action remains extremely limited. Considering, LPS may trigger cardiac dysfunction by causing mitochondrial injury; the current study builds upon our previous data allowing to hypothesize that EDPs protect cells by protecting mitochondrial homeostasis. Recent evidence demonstrates a crucial role for SIRT1 and SIRT3 in promoting mitochondrial biogenesis, function and mitohormesis.^12,13^ As well, activation of SIRT1 has been shown to confer protection against various pathophysiological conditions.^14,15^ We investigate whether EDP-associated enhancement and preservation of cardiac mitochondrial quality requires activation of SIRT1 signaling to reduce LPS-induced cytotoxicity.

## Results

### EDPs prevented LPS-induced morphological abnormalities in HL-1 cardiac cells

**Figure 1:**
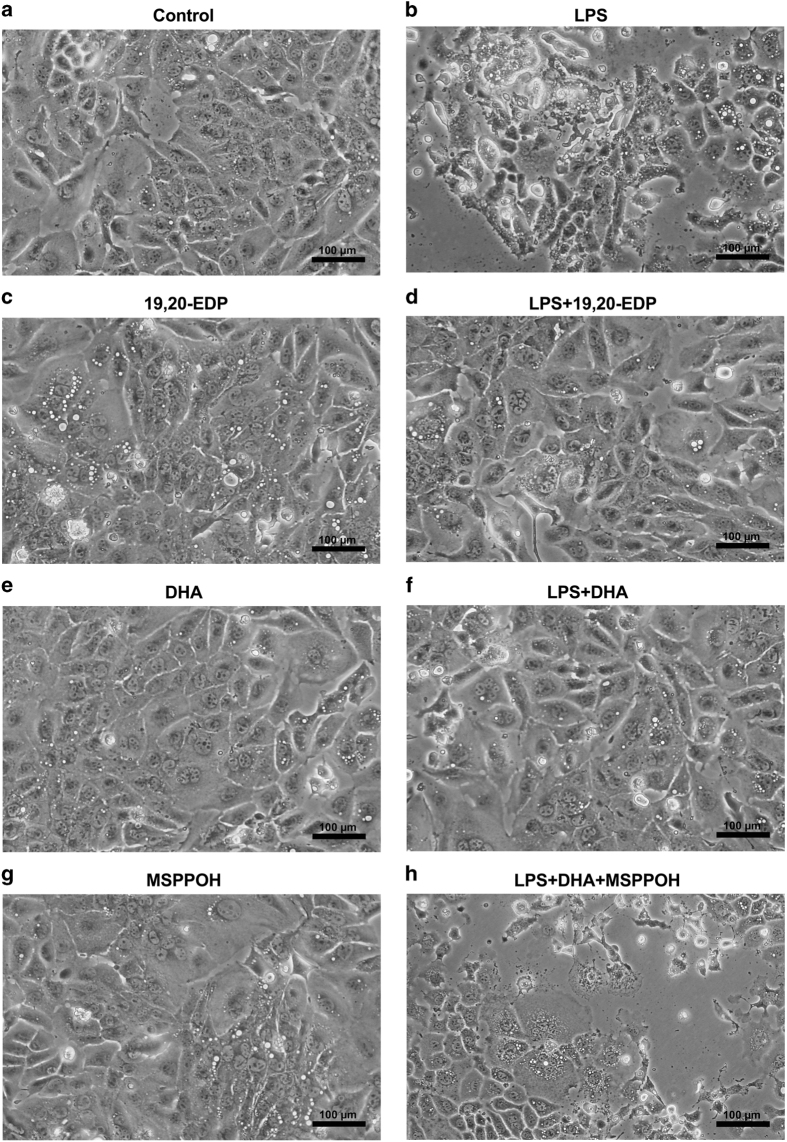
EDPs prevent LPS-induced morphological abnormalities in HL-1 cardiac cells. HL-1 cells were visualized by phase-contrast microscopy at 200× following treatment. (**a**) Untreated HL-1 cardiac cells. (**b**) HL-1 cardiac cells were stimulated with LPS (1 *μ*g/ml) for 24 h. (**c**) HL-1 cells were treated with 19,20-EDP (1 *μ*M) for 24 h. (**d**) HL-1 cells were treated with LPS (1 *μ*g/ml) and 19,20-EDP (1 *μ*M) for 24 h. (**e**) HL-1 cardiac cells were treated with DHA (100 *μ*M) for 24 h. (**f**) HL-1 cardiac cells were stimulated with LPS (1 *μ*g/ml) in the presence of DHA (100 *μ*M) for 24 h. (**g**) HL-1 cardiac cells were treated with MSPPOH (50 *μ*M) for 24 h. (**h**) HL-1 cardiac cells were stimulated with LPS (1 *μ*g/ml) in the presence of DHA (100 *μ*M) and MSPPOH (50 *μ*M) for 24 h. Assessment of cell morphology was performed on a minimum of 15 cells per treatment. Scale bar, 100 *μ*m in diameter.

Evaluation of morphological alterations using an inverted phase-contrast microscope revealed that HL-1 cells exposed to LPS for 24 h displayed marked morphological abnormalities such as formation of large vacuoles, shrinkage, rounding up and detachment from the surface of the plate (Figures 1a and b). The observation of cells demonstrating organelle swelling, ruptured membranes and partial lysis suggests there was more than one type of cell death occurred. This is consistent with the literature indicating TNF*α* may trigger both apoptosis and necroptosis.^16^ Treatment with 19,20-EDP did not alter the morphology of HL-1 cardiac cells (Figure 1c). Interestingly, we observed that co-treatment with 19,20-EDP prevented LPS-produced morphological abnormalities in HL-1 cells indicating a profound protective potential (Figure 1d). Treatment with DHA was without effect on morphology of HL-1 cardiac cells (Figure 1e). Cells co-treated with LPS and DHA showed a significant protection of morphology against LPS-induced injury (Figure 1f). In order to determine whether protective effects of DHA were mediated by its epoxy metabolites, we added N-(methylsulfonyl)-2-(2-propynyloxy)-benzenehexanamide (MSPPOH) to see if we could prevent DHA-induced protection. MSPPOH alone did not affect cell morphology (Figure 1g) but abolished DHA-associated protection (Figure 1h), suggesting a key role of EDPs but not DHA in the cytoprotective response against LPS-induced toxicity.

### EDPs protect HL-1 cardiac cells against LPS-induced injury

**Figure 2:**
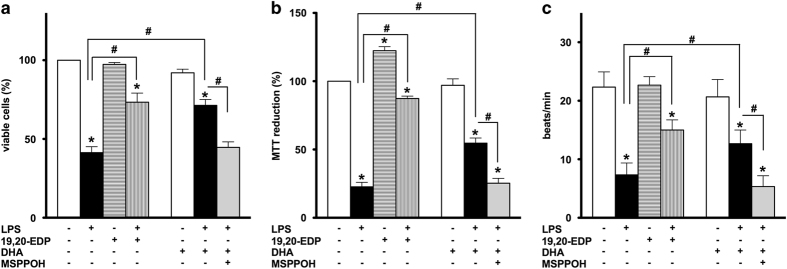
EDPs prevent LPS-induced cell death in HL-1 cardiac cells. HL-1 cardiac cells were stimulated with LPS (1 *μ*g/ml) in the presence of 19,20-EDP (1 *μ*M), DHA (100 *μ*M) and/or MSPPOH (50 *μ*M) for 24 h. (**a**) Cell viability was scored by trypan blue exclusion assay. (**b**) Mitochondrial activity was evaluated by MTT assay. (**c**) Contractility of HL-1 cells was scored by counting the number of beats per minute in five different cell clusters. Values are represented as mean±S.E.M. *N*=3 independent experiments. *P<0.05 treatment *versus* vehicle control; ^#^ *P*<0.05 treatment group *versus* LPS or LPS/MSPPOH.

Treatment with LPS resulted in a significant decline in cell viability as detected by a trypan blue exclusion assay and impaired mitochondrial oxidative activity evaluated by the MTT assay. These observations indicate disruption of mitochondrial function occurred in HL-1 cells in response to LPS resulting in a decreased number of viable cells (Figures 2a and b). The ability of cardiomyocytes to contract *in vitro* reflects their functional activity and requirement for a continuous supply of ATP to sustain optimum contractility.^17^ Considering mitochondria are the major site of ATP production in cardiomyocytes, disrupted contraction of HL-1 cardiac cells observed in our experiments suggests increased mitochondrial dysfunction occurred following LPS treatment (Figure 2c). We demonstrate that co-treatment with 19,20-EDP conferred significant protection to HL-1 cells exposed to LPS. Our data illustrate addition of 19,20-EDP greatly improved the viability of HL-1 cells and attenuated LPS-induced detriments to mitochondrial oxidative metabolism resulting in preserved contractility (Figure 2c).

Numerous *in vivo* and *in vitro* studies have provided strong evidence that DHA produces diverse and profound cardioprotective effects. Importantly, DHA may be metabolized by CYP epoxygenases into active epoxylipid metabolites, EDPs.^9^ Thus, we examined whether or not the protective effects of DHA are mediated by endogenously produced EDPs. In a parallel series of experiments, we treated LPS-exposed HL-1 cells with DHA or DHA plus MSPPOH to pharmacologically inhibit CYP epoxygenase activity and block formation of endogenous EDPs. DHA treatment significantly improved cell viability and functional activity of HL-1 cells exposed to LPS. Protective effects produced by DHA were abolished by co-treatment with MSPPOH, which highlights a crucial role of endogenously produced EDPs from exogenously added DHA in protection against LPS-induced cytotoxicity (Figures 2a and c). Collectively, our results suggest a key role for the epoxy metabolites of DHA, EDPs, in triggering protective responses in HL-1 cells against LPS-induced cytotoxicity.

### EDPs attenuate LPS-induced inflammation and cellular dysfunction

**Figure 3:**
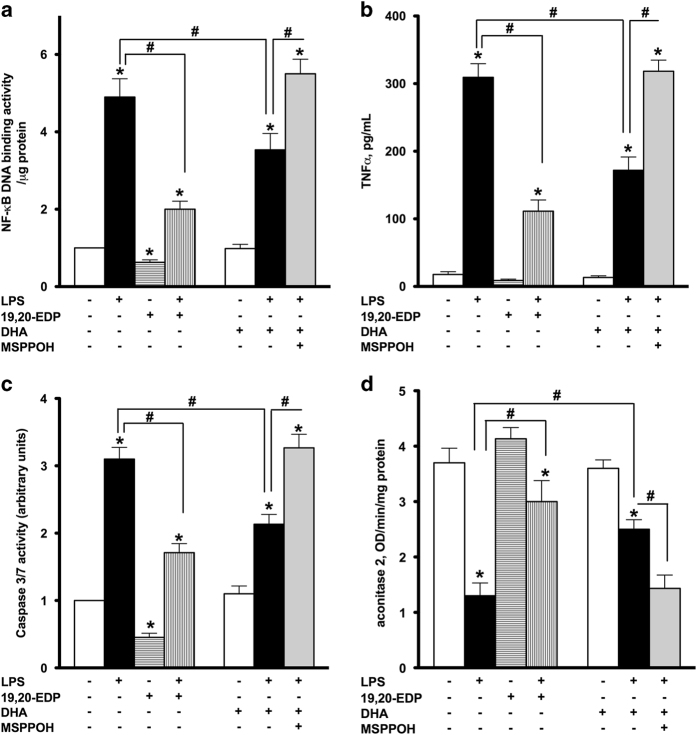
EDPs suppress LPS-induced inflammatory response, activation of caspase-3/7 and inhibition of aconitase 2. HL-1 cardiac cells were treated with LPS (1 *μ*g/ml) in the presence of 19,20-EDP (1 *μ*M), DHA (100 *μ*M) and/or MSPPOH (50 *μ*M) for 24 h. (**a**) NF-*κ*B DNA-binding activity in the whole-cell lysates was measured by ELISA. (**b**) TNF*α* concentration in the culture supernatants was determined by ELISA. (**c**) Caspase-3/7 activity was measured in the whole-cell lysates by spectrofluorometric assay. (**d**) Aconitase 2 activity was measured in the whole-cell lysates by colorimetric assay. Values are represented as mean±S.E.M. *N*=3 independent experiments. *P<0.05 treatment *versus* vehicle control; ^#^ *P*<0.05 treatment group *versus* LPS or LPS/MSPPOH.

In our previous studies, we demonstrated LPS triggered an inflammatory response in cardiomyocytes, which was partially associated with caspase activation and cell death.^18,19^ As expected, treatment with LPS promoted significant upregulation of NF-*κ*B DNA-binding activity initiating an inflammatory cascade (Figure 3a). Consistent with this observation, LPS caused release of the major pro-inflammatory cytokine TNF*α* from HL-1 cells (Figure 3b). Together, these results illustrate an inflammatory reaction developed in HL-1 cardiac cells exposed to LPS. Exposure to LPS also resulted in significant activation of caspase-3/7, indicative of an apoptotic response (Figure 3c). Aconitase 2 is an enzyme that catalyzes the reversible inter-conversion of citrate and isocitrate in the TCA cycle. Importantly, mitochondrial aconitase 2 also stabilizes mtDNA thereby influencing mitochondrial gene expression. A decrease in aconitase activity is considered a marker of mitochondrial and cellular damage.^20^ Exposure to LPS led to a pronounced decrease in aconitase activity in HL-1 cells (Figure 3d). Importantly, co-treatment with 19,20-EDP significantly attenuated the LPS-induced inflammatory response and attenuated activation of caspase-3/7 and aconitase 2 (Figures 3a–d). Although treatment with DHA attenuated LPS-associated detriments, it was less pronounced compared to 19,20-EDP. Moreover, this effect was sensitive to inhibition with MSPPOH, which further highlights the role of endogenously generated EDPs from DHA (Figures 3a–d).

### EDPs preserve the mitochondrial pool in HL-1 cells exposed to LPS

**Figure 4:**
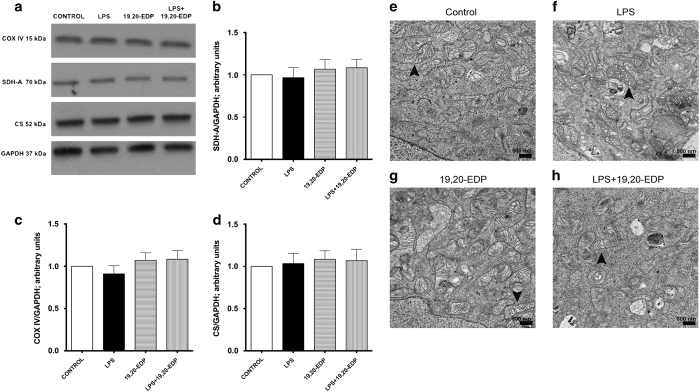
EDPs limit LPS-induced damage to mitochondrial ultrastructure. (**a**) HL-1 cardiac cells were stimulated with LPS (1 *μ*g/ml) with or without 19,20-EDP (1 *μ*M) where indicated for 24 h. After 24 h, the whole-cell lysates were harvested and then analyzed by western immunoblotting for the levels of essential mitochondrial proteins. Representative western blots and the results of quantification are demonstrated in (**a**–**d**). HL-1 cardiac cells were treated as indicated above. Representative electron micrograph (EM) images of HL-1 cells are presented on (**e**–**h**). Black arrowheads demonstrate individual mitochondrion. Scale bar, 500 *μ*m in diameter. Values are represented as mean±S.E.M. *N*=3 independent experiments.

First, we assessed the effects of LPS on the expression of essential mitochondrial proteins. No alterations were observed after all treatments suggesting that mitochondrial content was protected (Figures 4a–d). Next, we assessed mitochondrial ultrastructure using electron microscopy. Control HL-1 cardiac cells displayed normal mitochondrial morphology, where organization of mitochondria clearly displayed the internal membrane, cristae and well-defined outer membrane (Figure 4e). Treatment with LPS promoted marked structural heterogeneity in the mitochondrial population including patchy disruption of inner and outer membranes, formation of internal vacuoles, distorted cristae, clearer matrix and swelling. We also observed aberrant mitochondria of irregular size (Figure 4f). Treatment with 19,20-EDP did not alter mitochondrial ultrastructure (Figure 4g) but clearly limited LPS-induced injury of mitochondrial ultrastructure (Figure 4h). Overall our data suggest that pool of mitochondria found in HL-1 cells treated with 19,20-EDP was protected and thereby remained more functional.

### EDPs promote mitobiogenesis in HL-1 cells exposed to LPS

**Figure 5:**
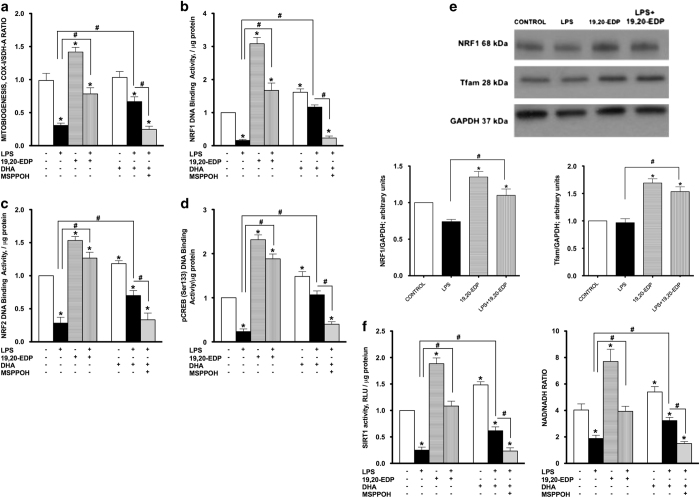
EDPs induce mitobiogenesis in HL-1 cardiac cells. HL-1 cardiac cells were stimulated with LPS (1 *μ*g/ml) in the presence of 19,20-EDP (1 *μ*M), DHA (100 *μ*M) and/or MSPPOH (50 *μ*M) for 24 h. (**a**) Relative rates of mitobiogenesis were assessed using ELISA detecting simultaneous expression of SDH-A (nDNA-encoded protein) and COX-I (mtDNA-encoded protein) in each well of plated HL-1 cells. The ratio between COX-I and SDH-A expressions represents the relative rate of mitobiogenesis. (**b**) NRF1 DNA-binding activity was measured in the whole-cell lysates using ELISA. (**c**) NRF2 DNA-binding activity was measured in the whole-cell lysates using ELISA. (**d**) pCREB (Ser133) DNA-binding activity was measured in the whole-cell lysates using ELISA. (**e**) Whole-cell lysates were harvested and then analyzed by western immunoblotting for the levels of key transcriptional regulators of mitobiogenesis. Representative western blots and the results of quantification are demonstrated. (**f**) SIRT1 activity was measured in the whole-cell lysates by bioluminescent assay in the presence of trichostatin A (1 *μ*M). And the levels of NAD and NADH were determined in the cells by bioluminescent assay. Values are represented as mean±S.E.M. *N*=3 independent experiments. *P<0.05 treatment *versus* vehicle control; ^#^ *P*<0.05 treatment group *versus* LPS or LPS/MSPPOH.

Initiating the generation of new mitochondria with improved bioenergetic efficiency is an important physiological strategy to increase production of ATP in response to stress conditions.^21^ Numerous studies have postulated that disruption of mitobiogenesis causes extensive cardiac dysfunction while activation, is thought to be cardioprotective.^22–24^ Regulation of mitobiogenesis occurs through the strict coordination of transcriptional factors such as NRF1, NRF2 and CREB.^23^ HL-1 cardiac cells exposed to LPS demonstrated a dramatic decline in overall mitobiogenesis based on the ratio between expression of the mitochondrial proteins COX-1 (mtDNA-encoded) and SDH-A (nDNA-encoded) measured simultaneously (Figure 5a). In order to further determine LPS effects on mitobiogenesis, we measured DNA-binding activities of key transcriptional regulators of mitobiogenesis. Our observations illustrate that LPS significantly reduced NRF1, NRF2 and pCREB (Ser133) DNA-binding activities, which suggests a negative impact on mitobiogenesis (Figures 5b and d). In a parallel series of experiments, we observed that treatment of healthy HL-1 cardiac cells with 19,20-EDP resulted in upregulation of mitobiogenesis as well as increased NRF1, NRF2 and pCREB (Ser133) DNA-binding activities (Figures 5b and d). Moreover, co-treatment of 19,20-EDP prevented the LPS-impaired mitobiogenesis (Figures 5a and d). Further, the increased NRF1 and Tfam protein expression levels following 19,20-EDP treatment were consistent with the observed increases in DNA-binding activities (Figure 5e). Although DHA produced a similar effect toward mitobiogenesis, inhibition of CYP epoxygenases with MSPPOH suppressed the response (Figures 5a and d). Together, these results suggest a key role for EDPs in promoting and regulating mitobiogenesis.

### EDPs prevent LPS-associated decrease in SIRT1 activity and NAD^+^ levels

An important regulator of mitobiogenesis and mitochondrial function is the histone deacetylase sirtuin-1 (SIRT1).^25^ SIRT1 activates PGC1*α* signaling to promote mitobiogenesis as well as promotes mitophagy to selectively eliminate severely damaged mitochondria.^26^ NAD^+^ is a crucial activator of SIRT1, therefore alterations in NAD/NADH ratios will significantly affect SIRT1 activity and function.^25^ As SIRT1 positively regulates mitochondrial quality, we examined whether 19,20-EDP could modulate SIRT1 activity and affect NAD/NADH ratios in HL-1 cells exposed to LPS. Treatment with LPS markedly decreased SIRT1 activity and lowered the NAD/NADH ratio in HL-1 cells (Figure 5f). Treatment with 19,20-EDP robustly activated SIRT1 activity and elevated the NAD/NADH ratio under control conditions, and prevented the decrease caused by LPS (Figure 5f). Similar protective results were observed when LPS-exposed HL-1 cells were co-treated with DHA and were abolished by the addition of MSPPOH.

### EDPs protect mitochondrial function in HL-1 cells exposed to LPS

**Figure 6:**
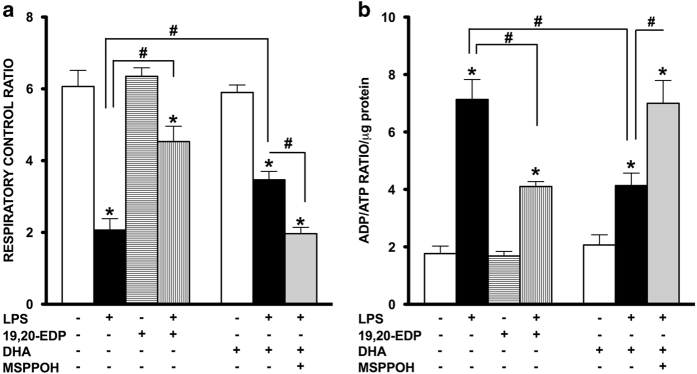
EDPs preserve mitochondrial function following LPS-induced cytotoxicity. HL-1 cardiac cells were stimulated with LPS (1 *μ*g/ml) in the presence of 19,20-EDP (1 *μ*M), DHA (100 *μ*M) and/or MSPPOH (50 *μ*M) for 24 h. (**a**) Cells were harvested and transferred into Clark-electrode-based chamber connected to Oxygraph at 30 °C. Rates of respiration were measured in saponin-permeabilized cells using 10 mm glutamate and 5 mM malate as substrates. ADP-stimulated respiration was measured after addition of 1 mM ADP. The rates of respiration are expressed as respiratory control ratio (RCR). (**b**) The intracellular ratio between ADP and ATP was measured by chemolumenescent assay and normalized per *μ*g of protein. Values are represented as mean±S.E.M. *N*=3 independent experiments. *P<0.05 treatment *versus* vehicle control; ^#^ *P*<0.05 treatment group *versus* LPS or LPS/MSPPOH.

The most essential component of mitochondrial function is respiration coupled with generation of ATP also referred as oxidative phosphorylation. The ratio between oxygen consumption by mitochondria in basal and ADP-stimulated states indicates respiratory control ratio (RCR), which reflects bioenergetic efficiency of mitochondria.^27^ We measured mitochondrial respiration in permeabilized HL-1 cells to characterize the effects of LPS and EDPs on mitochondrial function, where the organelles exist within their native intracellular localization. Treatment with LPS caused a significant decrease in mitochondrial RCR, reflecting the overall collapse in mitochondrial function (Figure 6a), whereas addition of 19,20-EDP prevented the LPS-mediated mitochondrial dysfunction. Paralleling our observations with regard to compromised mitochondrial function, we demonstrate that exposure to LPS markedly increased the ADP/ATP ratio in the cells providing further support that mitochondrial function was severely compromised (Figure 6b). Treatments with 19,20-EDP and DHA resulted in a significant reduction of LPS-induced elevation in the ADP/ATP ratio. Consistently, similar protective results were observed when LPS-exposed HL-1 cells were co-treated with DHA and abolished by the addition of MSPPOH (Figures 6a and b).

### Pharmacological inhibition of SIRT1 activity reverses 19,20-EDP-mediated protection against LPS-induced cytotoxicity

**Figure 7:**
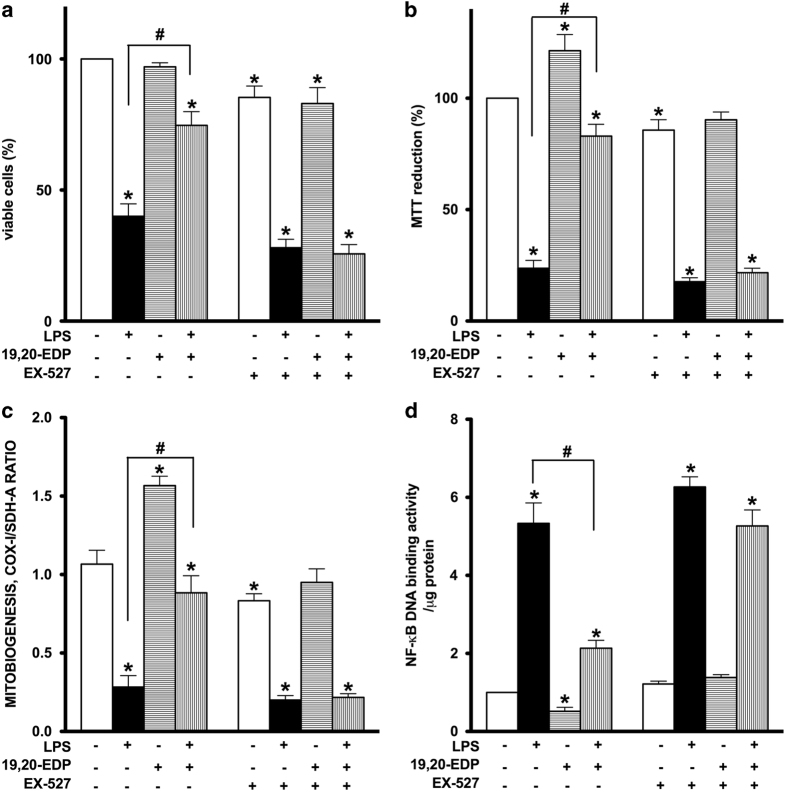
Inhibition of SIRT1 activity abolished 19,20-EDP protective effects against LPS-induced cytotoxicity. HL-1 cardiac cells were stimulated with LPS (1 *μ*g/ml) in the presence of 19,20-EDP (1 *μ*M) and EX-527 (1 *μ*M) for 24 h. (**a**) Cell viability was scored by trypan blue exclusion assay. (**b**) Mitochondrial activity was measured by MTT assay. (**c**) The relative rates of mitobiogenesis were assessed using ELISA detecting simultaneous expression of SDH-A (nDNA-encoded protein) and COX-I (mtDNA-encoded protein) in each well of plated HL-1 cells. The ratio between COX-I and SDH-A expressions represents the relative rate of mitobiogenesis. (**d**) NF-*κ*B DNA-binding activity in the whole-cell lysates was measured by ELISA. Values are represented as mean±S.E.M. *N*=3 independent experiments. *P<0.05 treatment *versus* vehicle control; ^#^ *P*<0.05 treatment group *versus* LPS.

To determine if SIRT1 may be mediating 19,20-EDP-associated effects, we treated HL-1 cardiac cells with EX-527 a specific inhibitor of SIRT1. Treatment with EX-527 abolished the protective effects of 19,20-EDP, whereas inhibition of SIRT1 prevented 19,20-EDP-associated improvements in cell viability and mitochondrial activity in cells treated with LPS (Figures 7a and b). Furthermore, inhibition of SIRT1 prevented 19,20-EDP from triggering mitobiogenesis and its ability to limit LPS-induced impairments (Figure 7c). Importantly, pharmacological inhibition of SIRT1 restored NF-*κ*B DNA-binding activity, which was suppressed by 19,20-EDP (Figure 7d). Together, our observations suggest an essential role for SIRT1 in 19,20-EDP-mediated protective effects against LPS-induced cytotoxicity.

## Discussion

CYP epoxygenases catalyze the enzymatic transformation of PUFA into biologically active epoxylipid mediators, such as omega-6 epoxides of AA, EETs and omega-3 epoxides of DHA, EDPs.^9^ Although there is strong evidence demonstrating the biological effects of EETs, there is little known regarding EDP-mediated effects. Emerging evidence suggests that EDPs are involved in regulating numerous aspects of cell biology such as inflammation, angiogenesis and cell death; however, our understanding how EDPs regulate these effects remains significantly limited.^19^

In the current study, we report EDP-mediated events protect HL-1 cardiac cells against LPS-induced cytotoxicity by maintaining a healthy pool of mitochondria. We demonstrate that EDPs significantly attenuate mitochondrial damage attributed to LPS exposure and promote the generation of new mitochondria by activating biogenesis. Our data suggest EDPs work by activating SIRT1 signaling to promote an adaptive response and counteract deleterious effects of LPS. Furthermore, we demonstrate that the protective effect of DHA against LPS-induced cytotoxicity requires its metabolic transformation catalyzed by CYP epoxygenase into endogenous EDPs.

The preservation of mitochondrial ultrastructure and function as a result of co-treatment of 19,20-EDP with LPS demonstrates the ability of EDPs to preserve and maintain mitochondrial quality. These data suggest the mitochondrial pool in the LPS-treated groups was severely compromised compared to EDP-treated groups. This observation is consistent with our previously published data where we demonstrated mitochondrial quality was protected with increased levels of epoxylipids during starvation.^28^ Optimally functioning mitochondria require a balanced control of biogenesis and elimination to maintain a healthy pool of organelles that provide energy, regulate cell survival/death pathways and synthesize a number of biologically active molecules. Several lines of evidence suggest maintaining mitochondrial homeostasis and integrity is directly linked to cellular protection under stress conditions.^17^ As cardiomyocytes are terminally differentiated cells, sustaining functional mitochondria is important to promote adequate cell survival in the setting of cardiac dysfunction. LPS treatment has previously been demonstrated to induce extensive injury of cardiac mitochondria resulting in cell death and overall cardiac dysfunction.^29^ Consistent with the literature, our data demonstrated LPS-mediated cardiotoxicity had a large impact on mitochondrial quality culminating in extensive damage associated with cell death. Therefore, we hypothesize that EDPs can enhance mitochondrial quality control by regulating function, biogenesis and turnover, thereby sustaining a healthier pool of mitochondria.

Regulation of mitochondrial quality is a complex process that involves a strictly coordinated interaction between mitochondrial and nuclear genomes orchestrated by NRF1/2, Tfam, SIRT1/3, CREB and PGC1*α* pathways.^17^ An important outcome is the generation of new organelles that efficiently supply the cell with its required amounts of ATP.^24^ Our results demonstrating improved ADP/ATP ratio and increased mitochondrial O_2_ consumption following 19,20-EDP treatment reflect significantly improved mitochondrial function. Moreover, treatment with 19,20-EDP stimulated the activity of key factors required for activation of mitobiogenesis suggesting production of new organelles. Specifically, we showed 19,20-EDP upregulated the transcriptional activity of CREB and NRF1/2 as well as increased enzymatic activity of SIRT1. Collectively, our data indicate EDP-mediated effects, which activate mitobiogenesis as an inherent part of the overall protective response against LPS-induced cytotoxicity.

CREB is an essential transcriptional factor required for regulating mitobiogenesis, where phosphorylation of CREB promotes a rapid and robust activation cascade of reactions evoking mitobiogenesis.^30^ In this study, 19,20-EDP caused a strong increase in pCREB (Ser133) DNA-binding activity, which suggests an involvement of CREB-dependent pathways in the observed effects. Activated CREB directly interacts with other key factors of mitobiogenesis such as NRF1/2 and SIRT1. Remarkably, CREB can translocate from the cytosol to both the nucleus and inner mitochondrial membrane where it acts as a positive regulator of mitochondrial function.^30^ In the current study, we assessed pCREB nuclear DNA-binding activity but it is unclear if there is a role for mitochondrial localization. Interestingly, a recent study demonstrated another epoxylipid of CYP expoxygenase, 14,15-EET, protected neurons against ischemia-reperfusion insult by promoting mitobiogenesis through activation of CREB-dependent pathways.^31^

SIRT1 has received significant attention as a crucial regulator of mitobiogenesis and function.^25^ Enzymatic reactions catalyzed by SIRT1 require the cofactor NAD^+^ to deacetylate a wide range of proteins including PGC1*α*. SIRT1-dependent deacetylation of PGC1*α* has been shown to trigger mitobiogenesis as an adaptive reaction to various pathophysiological factors, such as starvation, disrupted glucose homeostasis, oxidative stress, aging and cardiovascular disease.^14^ SIRT1, SIRT3 and SIRT6 can enhance mitochondrial function by activating oxidative phosphorylation and suppressing glycolysis.^15,32^ Genetic overexpression of SIRT1 resulted in positive metabolic outcomes,^33,34^ whereas SIRT1-deficient mice have a shortened lifespan with a pronounced metabolic defect.^35^ Furthermore, a direct piece of evidence indicates SIRT1 triggers the deacetylation of essential autophagic proteins promoting the selective removal of damaged mitochondria through mitophagy in human fibroblasts, which ultimately results in positively modulating quality.^26^ Together, these studies support a concept where activation of SIRT1 is required to enhance and sustain optimal mitochondrial quality through promoting biogenesis and simultaneous removal of damaged mitochondria. This dual positive regulatory role SIRT1 has toward mitochondrial quality results in selecting and maintaining a highly functional pool of mitochondria. Interestingly, Yeung *et al*.^36^ demonstrated the ability of SIRT1 to deacetylate and consequently inactivate NF-*κ*B, a major factor orchestrating pro-inflammatory responses. This provides evidence that SIRT1 can limit the inflammatory injury to mitochondria and illustrates its protective role. Our study also demonstrates the importance of SIRT1 in limiting NF-*κ*B-dependent inflammation in cardiac cells exposed to LPS. The fact pharmacological inhibition of SIRT1 abruptly terminated EDP-mediated protection supports the notion for a key role of SIRT1 signaling. Whether EDP-associated activation of SIRT1 occurs directly or requires involvement of secondary pathways remains currently unknown. However, the overarching result demonstrates EDPs promote and sustain an optimally functioning population of mitochondria.

During the last decade, studies concerning the favorable biological effects of DHA have largely ignored the roles of CYP-derived metabolites. In this study, we demonstrate that DHA produces a protective response in HL-1 cells exposed to LPS similar to what was observed with 19,20-EDP. Particularly, our data highlight DHA treatment preserved cell viability, robustly stimulated mitochondrial biogenesis and function against LPS-induced cytotoxicity. Recent studies demonstrate protective effects of DHA, including improved vascular function and reduced NF-*κ*B-dependent polarization of macrophages, are mediated through SIRT1.^37,38^ Our current study demonstrates the effects produced by DHA are abolished by MSPPOH, a specific inhibitor of CYP epoxygenase, which prevents the endogenous production of EDPs. Thus, suggesting the biological effects of DHA are attributed to CYP epoxygenase metabolites, most likely the epoxylipids, EDPs, and not DHA itself.

**Figure 8:**
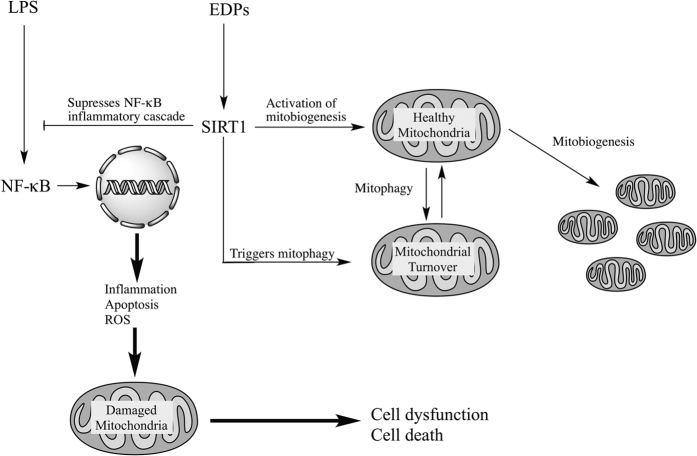
Schematic showing activation of SIRT1 activity by EDPs is required to exert protective effects. LPS exposure activates a NF-*κ*B-dependent inflammatory response triggering extensive mitochondrial injury with associated decreased quality control. Subsequently, the compromised mitochondria rapidly promote cell dysfunction and cell death. EDPs act as positive and possibly, selective modulators of SIRT1 activity, through yet to be identified molecular mechanisms, initiating important adaptive responses. SIRT1 can (i) act as a potent suppressor of NF-*κ*B; (ii) rapidly and potently activate mitobiogenesis; and (iii) selectively eliminate damaged mitochondria via mitophagy. EDP-mediated activation of SIRT1 signaling promotes physiological events that enhance mitochondrial quality control. Thus, preserving a healthy and optimally functioning pool of mitochondria, which protect the cell from LPS-induced toxicity.

In summary, our results demonstrate the CYP epoxygenase metabolites of DHA, EDPs, preserve the pool of mitochondria in HL-1 cardiac cells limiting LPS-induced cytotoxicity. Although the precise molecular mechanisms remain unknown, we propose that EDP-mediated effects require activation of SIRT1 signaling to initiate events to promote mitobiogenesis and enhance function. This cascade of adaptive reactions can be considered a response directed to maintain a healthy mitochondrial pool and thereby promote cell survival (Figure 8).

## Materials and Methods

### Cell culture

HL-1 cardiac cells were a kind gift from Dr. Claycomb (New Orleans, USA). Cells were cultivated in Claycomb media supplemented with glutamine and norepinephrine as described.^28^ HL-1 cells were maintained at 37 °C in a humidified atmosphere of 5CO_2_ and 95% air. Cell viability was assessed by using trypan blue exclusion test as previously described.^28^ Beating rate was estimated by counting the number of beats per minute in five different cell clusters in five independently blinded experiments.

### Treatment protocols

HL-1 cells were exposed to LPS (1 *μ*g/ml) for 24 h. Where indicated, cells were also treated or co-treated with the following pharmacological agents: 19,20-EDP (1 *μ*M), DHA, metabolic precursor of endogenous EDPs (100 *μ*M), MSPPOH (50 *μ*M) to specifically inhibit CYP epoxygenase activity and block formation of endogenous EDPs from DHA and EX-527 (1 *μ*M) to selectively inhibit SIRT1 activity. Stock solutions of 19,20-EDP, DHA, MSPPOH and EX-527 were prepared in 100% ethanol; final concentrations of both solvents were <0.01% of the treatment solutions. LPS and EX-527 were purchased from Sigma-Aldrich (Oakville, ON, Canada). 19,20-EDP, DHA and MSPPOH were obtained from Cayman Chemical (Ann Arbor, MI, USA).

### Assessments of mitochondrial function and biogenesis

In order to test overall efficiency of mitochondrial oxidative metabolism, we measured the ADP/ATP ratio in cell lysates using a luciferase-based method (Sigma-Aldrich). NAD/NADH ratio was assessed using bioluminescent kit (Promega, Madison, WI, USA). An MTT assay was used to examine total oxidative metabolism as previously described.^28^ The intensity of reduction of 3-(4,5-dimethylthiazol-2-yl)-2,5-diphenyltetrazolium bromide to formazan crystals by mitochondrial dehydrogenases positively correlates with the overall activity of oxidative metabolism.^39^ Optical density of DMSO extracted formazan was measured spectrophotometrically at 595 nm. Mitochondrial respiration was measured in saponin-permeabilized HL-1 cells using Clark oxygen electrode connected to Oxygraph Plus recorder (Hansatech Instruments Ltd., Norfolk, England).^27^ Respiration rates were measured at 30 °C before and after addition of 2 mM ADP with 5 mM malate and 10 mM glutamate as substrates. RCR was calculated as the ratio between basal and ADP-stimulated respiration rates. Mitobiogenesis was evaluated using an ELISA kit (Abcam, Cambridge, UK) based on simultaneous detection of SDH-A, a subunit of complex II (nDNA-encoded protein) and COX-1, a subunit of complex IV (mtDNA-encoded). The ratio between these proteins reflects the intensity of mitobiogenesis.

### Caspase-3/7, SIRT1 and aconitase 2 activity assays

Caspase-3/7 activity in cardiomyocytes was detected by using the Apo-ONE and SIRT1 enzymatic activity by SIRT-Glo assay kits (Promega) according to the manufacturer’s instructions. Aconitase 2 (mitochondrial isoform) activity as a marker of mitochondrial damage was evaluated using an ELISA kit (Abcam).

### Cytokine assay

The medium was centrifuged for 5 min at 5000×*g* and supernatants were analyzed by ELISA for TNF*α* levels (Abcam). Briefly, the sample was added into individual wells of a 96-well plate coated with a TNF*α* mouse-specific antibody. After washing, wells were incubated with HRP-conjugated streptavidin, washed and incubated with substrate solution. The intensity of the color was measured spectrophotometrically at 450 nm. Increased color intensity occurred in a linear proportion to the amount of TNF*α* in the samples.

### NRF1, NRF2, pCREB (Ser133) and NF-*κ*B DNA-binding assays

NRF1 DNA-binding assay was performed using an ELISA kit from Assay Biotech (Sunnyvale, CA, USA), pCREB (Ser133) DNA-binding activity was measured using an ELISA kit from Cayman Chemical, NRF2 and NF-*κ*B DNA-binding assays were performed using ELISA kits from Active Motif (Carlsbad, CA, USA).

### Western blot assay and antibodies

HL-1 cells were treated as described above, harvested and the whole-cell lysates were prepared and subjected to western blot assay as previously described.^11^ Samples were probed with antibodies against NRF1, SDH-A, Tfam, COX IV, GAPDH (Cell Signaling Technology, Boston, MA, USA) and CS (Abcam). Quantitation was performed using using ImageJ software (NIH, Bethesda, MD, USA).

### Microscopy

HL-1 cells were grown on coverslips and then treated as described above for 24 h. Cells were then fixed with 2% glutaraldehyde in 0.1 M sodium cacodylate for 30 min. Cell monolayer was then post-fixed in 1% sodium tetroxide in 0.1 M sodium cacodylate for 30 min on ice and in the dark. Approximately, 2% uranyl acetate was used for en bloc staining of the samples for 30 min on ice and in the dark. Dehydration was done by exposing the samples to increasing concentrations of ethanol (50–100%). Finally, resin-filled beams were transferred upside-down on top of the cells and left at 60 °C in a incubator for 48 h to polymerize. Imaging was done by a Philips 410 electron microscope (Toronto, ON, Canada), using MegaView III soft imaging system (Richmond Hill, ON, Canada) and ITEM software (Irvine, CA, USA). Morphological evaluation of HL-1 cells was performed after incubation for 24 h with indicated agents using an inverted phase-contrast microscope (Olympus CKX41 with Optika Pro5 camera, Richmond Hill, ON, Canada).

### Statistical analysis

Data are presented as mean±S.E.M. Statistical analysis was based on one-way ANOVA with a Bonferroni *post hoc* test; *P*<0.05 was considered statistically significant.

## Abbreviations

AA: arachidonic acid
CS: citrate synthase
CYP: cytochrome *P*450
DHA: docosahexaenoic acid
EDPs: epoxydocosapentaenoic acids
LPS: lipopolysaccharide
MSPPOH: *N*-(methylsulfonyl)-2-(2-propynyloxy)-benzenehexanamide
NF-*κ*B: nuclear factor kappa B
PUFAs: polyunsaturated fatty acids
RCR: respiratory control ratio
sEH: soluble epoxide hydrolase
SIRT1: sirtuin-1
TNF*α*: tumor necrosis factor.
